# Urachal Signet Ring Cell Carcinoma: A Clinicopathological Analysis of 28 Cases

**DOI:** 10.3390/curroncol33030153

**Published:** 2026-03-07

**Authors:** Natalie South, Ioana Maria Mihai, Vickie Wang, Mehdi Agoumi, Charles Guo, Gang Wang

**Affiliations:** 1 School of Medicine, University of Medicine and Health Sciences, Royal College of Surgeons in Ireland, D02 YN77 Dublin, Ireland; 2 Department of Pathology and Laboratory Medicine, BC Cancer, Vancouver, BC V6T 1Z1, Canada; 3 Department of Pathology and Laboratory Medicine, University of British Columbia, Vancouver, BC V6T 1Z7, Canada; 4 Faculty of Science, University of British Columbia, Vancouver, BC V6T 1Z4, Canada; 5 Department of Pathology and Laboratory Medicine, Surrey Memorial Hospital, Surry, BC V3V 1Z2, Canada; 6 Department of Pathology, MD Anderson Cancer Center, Houston, TX 77030, USA

**Keywords:** urachus, carcinoma, bladder, urachal signet-ring cell carcinoma, case series

## Abstract

**Background:** Urachal carcinoma is a rare malignancy comprising less than 1% of all bladder cancers. The signet-ring cell subtype is particularly aggressive and poses significant diagnostic and therapeutic challenges. **Methods:** A retrospective review of urachal carcinoma cases from 1989 to 2023 was conducted using data from BC Cancer and MD Anderson Cancer Center. The study analyzed 75 patients, including 28 signet-ring cell carcinoma (SRCC) cases and a control group of 47 non-SRCC cases, to compare survival patterns, treatment outcomes, and histopathological features. **Results:** Clinically, the SRCC subtype was associated with advanced stage at presentation (pT3/pT4) and a higher recurrence rate (82% vs. 53%; *p* = 0.01). Survival analysis demonstrated worse outcomes for the SRCC cohort, with a five-year cancer-specific survival (CSS) of 39% compared to 64% in the non-signet group (*p* = 0.053). Partial cystectomy remained the primary surgical approach for both cohorts. Adjuvant chemotherapy was administered more often in SRCC cases (86% vs. 47%). **Conclusions:** By providing a comprehensive multi-institutional analysis, this study establishes urachal SRCC as a distinct clinical entity with a strong independent prognostic significance. Despite aggressive multimodal management, it carries a poorer prognosis compared to urachal non-SRCC, emphasizing the need for subtype-specific therapeutic guidelines.

## Simple Summary

Urachal carcinoma of the bladder is a rare malignancy that develops from urachal remnants. The signet ring cell subtype is a particularly rare and aggressive subtype that often presents a diagnostic challenge. This study analyzed a multi-institutional cohort of 75 patients, comparing 28 cases of urachal signet ring cell carcinomas with 47 cases of different urachal carcinomas subtypes. Our results show that the signet ring cell subtype is associated with advanced disease at diagnosis and a higher recurrence rate compared to the control group. Despite aggressive surgical management and chemotherapy, patients with this subtype had lower survival rates. Overall, the presence of signet ring cells is a strong independent predictor of poor prognosis. These results highlight the importance of implementing dedicated clinical strategies and therapeutic interventions targeted to this specific histological profile.

## 1. Introduction

Urachal carcinoma is a rare malignancy originating from urachal remnants, representing less than 1% of all bladder cancers [1,2,3]. The urachus is a tubular structure extending from the urinary bladder to the umbilicus during fetal development. Typically, the urachus regresses, but remnants persist in about one-third of individuals [2]. These remnants can undergo malignant transformation, leading to urachal carcinoma [4]. Urachal tumors are typically found at the bladder dome or anterior wall. Common sites of distant metastasis include the lungs, lymph nodes, bones, brain, and liver [5]. Urachal cancer, although rare, has a uniformly poor prognosis, in part due to the challenges of diagnosing and treating it [3,6]. Approximately 90% of urachal cancers are glandular, while the remaining 10% are transitional, squamous, neuroendocrine, or mixed types [3,4,7,8]. A rare subtype of urachal carcinoma is the signet-ring cell. Morphologically, this subtype is characterized by extensive infiltrative growth of signet-ring cells within the stroma [9]. A signet ring cell component, admixed with other morphologies of urachal malignancies, accounts for 5–7% of urachal carcinomas [3,6,10]. Consequently, a true primary signet ring cell carcinoma (SRCC) of the urachus is extremely rare [11,12,13,14,15,16,17,18,19,20,21,22]. The presence of signet ring cells has been identified as a very aggressive subtype, associated with a poor prognosis [20]. More often, signet ring cells are more commonly observed in the urachus and bladder as a result of metastasis from other primary sites, primarily of gastrointestinal origin [23]. Research on this subtype is limited, and established treatment guidelines are lacking [3,6,24]. This study aims to examine the survival patterns of urachal SRCC, current treatment modalities, and histopathological characteristics of urachal SRCC, based on a large, multi-institutional database.

## 2. Materials and Methods

### 2.1. Patients

We conducted a retrospective review from a combined pathology database to find urachal SRCC cases diagnosed between 1989 and 2023. This database included cases from the British Columbia (BC) Pathology database and from the MD Anderson database.

We reviewed medical charts to gather data on patient demographics, clinical history, tumor location, gross pathological findings, and morphological characteristics. Medical records were also reviewed to collect information on the patient’s treatment regimen, which included partial or radical cystectomies, radiation, and/or chemotherapy. Histological analysis was performed on hematoxylin and eosin (H&E)-stained slides retrieved from the archives. For diagnostic consistency, case selection was evaluated by two senior pathologists (C.G. and G.W.) at the two participating centers. To ensure a comparable group, we used the same combined pathology database from both BC Cancer and MD Anderson Cancer Center to select a control group with urachal bladder carcinomas that did not contain signet ring cells. In both study groups, patients presented similarly clinically at diagnosis, as well as in terms of stage and age at the time of diagnosis.

Tumor staging was evaluated using the American Joint Committee on Cancer TNM criteria [25]. Pathological staging was determined from either biopsy, transurethral resection of bladder tumor (TURBT) specimens, or cystectomy specimens, where available. However, due to the limitations of TURBT specimens and the absence of a radical or partial cystectomy for some patients, a uniform and accurate pT staging could not be applied to the entire cohort. Morphologically, the signet-ring cell subtype was defined by the presence of signet-ring cells showing widespread invasion into the surrounding stromal tissue [9].

### 2.2. Immunohistochemistry

We identified a subgroup of cases within the study that had previously undergone immunohistochemical (IHC) staining. This existing data was then incorporated into our analysis. The following monoclonal antibodies were used: GATA 3 (clone L50-823, 1:100 dilution: Cell Marque, Rocklin, CA, USA), CK7 (clones OV-TL 12/30 prediluted; Dako, Carpinteria, CA, USA), CK20 (clone Ks20.9, prediluted; Dako), CDX2 (1:200 dilution, Dako), SATB2 (clone EP281, prediluted, Cell Marque), and β-Catenin (clone 14, prediluted, Cell Marque). Standard tissue sections were deparaffinized in xylene and hydrated in graded alcohol. Immunostaining was performed using a DAKO autostainer (Agilent, Santa Clara, CA, USA). Slides were incubated with the primary antibody and then with a visualization reagent (secondary goat anti-mouse immunoglobulin and horseradish peroxidase linked to a dextran polymer backbone). The slides were then rinsed with distilled water, incubated with a 3,3-diaminobenzidine substrate-chromogen solution, and subjected to Mayer haematoxylin counterstaining [26]. Where available, IHC reports for mismatch repair (MMR) proteins were documented from the electronic database for MSH6 (clone EP49) and PMS2 (clone EP51).

### 2.3. Statistical Analysis

Statistical analysis was performed using R Statistical Software (version 4.5.2; R Core Team, 2025) within the RStudio (2025.05.1+513) Integrated Development Environment (Posit Software, PBC) [27]. The normality of distribution for continuous variables was assessed using the Shapiro–Wilk test. For the comparison of clinical and pathological characteristics and other morphologies, continuous variables were compared using the Wilcoxon rank-sum test, while categorical variables were analyzed using Pearson’s chi-squared test or Fisher’s exact test, as appropriate. Cancer-specific survival (CSS) and overall survival (OS) were estimated using the Kaplan–Meier method, and survival curves were compared using the log-rank test. OS was defined as the time from diagnosis to death of any cause or last follow-up, and CSS was defined as the time from diagnosis to death due to urachal malignancy. Multivariate analysis was performed using the Cox proportional hazards regression model to identify independent prognostic factors for both OS and CSS. Hazard ratios (HR) and 95% confidence intervals (CI) were calculated for each predictor. All tests were two-tailed, and a *p*-value < 0.05 was considered statistically significant.

## 3. Results

### 3.1. Clinical Characteristics

The study cohort included a total of 75 patients with primary urachal adenocarcinoma (Table 1). Of these, 28 cases (28/75, 37.3%) were classified as urachal SRCC, while the other 47 cases (47/75, 62.7%) with urachal carcinoma morphologies without signet-ring cell features served as a control group. Of the 28 SRCC cases, 21 (21/28, 75%) were male, and 7 (7/28, 25%) were female patients. The mean age at diagnosis was 53 years, and the median was 50.5 years. This compares to the control study group of 47 patients with urachal bladder carcinomas that did not contain signet ring cells (26 males [26/47, 55.3%], 21 females [21/47, 44.7%]), where the mean age at diagnosis was 54 years, and the median was 52.

The most frequent clinical presentation was gross or microscopic hematuria (31/75, 41.3%), observed in most of the symptomatic patients. Eight patients (8/75, 10.7%) presented with abdominal or pelvic pain, often localized to the suprapubic or infraumbilical region, or associated with a palpable mass. Umbilical abnormalities, including erythema, soreness, or swelling, were noted in three cases (3/75, 4%). Mucinuria was rarely reported (2/75, 2.7%). Notably, four patients (4/75, 5.3%) were asymptomatic at diagnosis, with tumors discovered incidentally during imaging or evaluation for unrelated conditions.

Initial diagnosis was confirmed on TURBT specimens in 48 cases (48/75, 64%) and on biopsy in 6 cases (6/75, 8%), and immediate partial or radical cystectomy was performed in 21 cases (21/75, 28%). Of the 54 patients (54/75, 72%) initially diagnosed by TURBT or biopsy, 37 (37/54, 68.5%) subsequently underwent radical or partial cystectomy. The remaining patients (17/54, 31.5%) were managed with systemic therapy or surveillance due to advanced disease or comorbidities.

Tumors were predominantly located at the bladder dome (65/75, 86.7%) or the anterior wall (10/75, 13.3%). Tumor size varied widely across the cohort, ranging from 0.5 cm to 10.5 cm in greatest dimension.

### 3.2. Pathological and Immunohistochemical Findings

**Figure 1:**
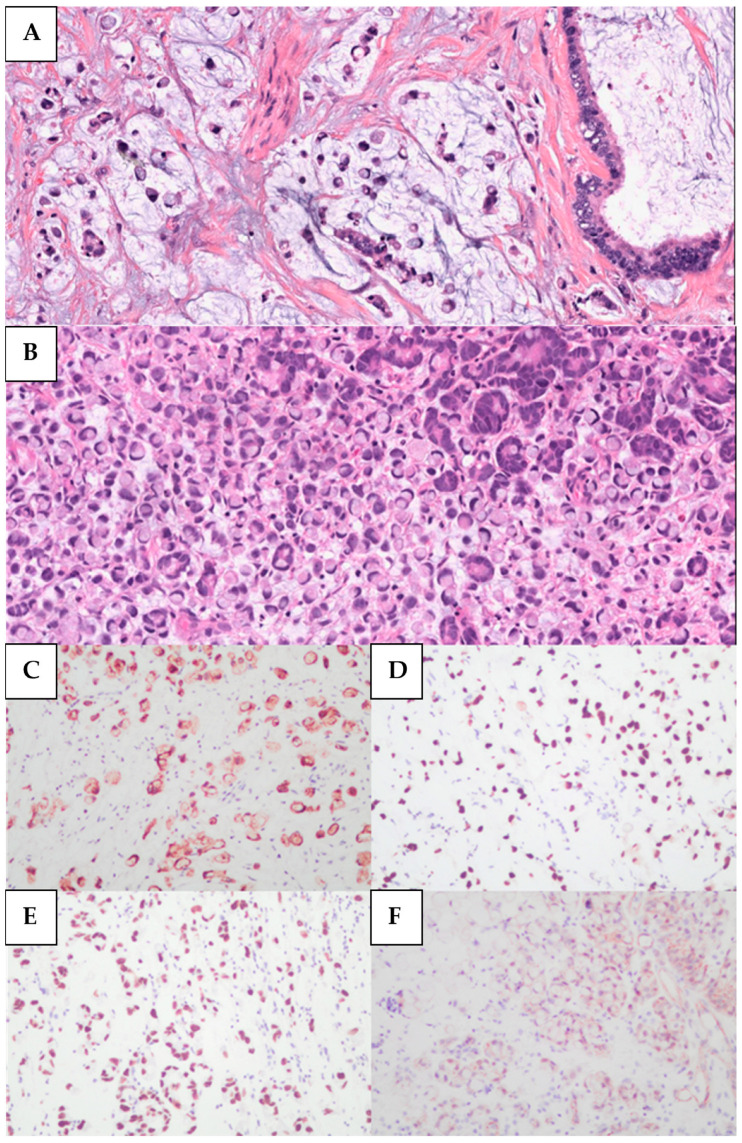
**Histopathology and immunohistochemistry of Urachal SRCC**. (**A**) Signet-ring cells and extracellular mucinous pools exhibiting permeative stromal growth, H&E, 200×. (**B**) Signet-ring cells with a diffuse architecture, H&E, 200×. (**C**) Signet-ring cells with positive cytoplasmic expression for CK20, 100×. (**D**) Signet ring cells with nuclear positive expression for CDX2, 100×. (**E**) Signet ring cell adenocarcinoma, positive nuclear staining for SATB2, 100×. (**F**) Signet ring cell adenocarcinoma with cytoplasmic staining for β-Catenin, 100×.

Morphologically, the 28 SRCC cases were characterized by a diffuse stromal infiltration of tumor cells displaying eccentric nuclei, abundant eosinophilic cytoplasm, and prominent cytoplasmic vacuoles (Figure 1). Pure signet-ring cell morphology was identified in 7 cases (7/28, 25%), while the remaining 21 cases (21/28, 75%) exhibited signet-ring cells within a background of mucin. The following histological subtypes of urachal carcinoma were included in the control cohort (n = 47): mucinous (23/47, 48.9%), enteric type (12/47, 25.5%), mixed mucinous and enteric (4/47, 8.5%), adenocarcinoma NOS (not otherwise specified) (6/47, 12.8%), and poorly differentiated (2/47, 4.3%).

Lymphovascular invasion (LVI) was observed in more than half of the SRCC cases (16/28, 57%), compared to less than a third of the control group (14/47, 30%). Urachal remnants were histologically identified in 15 cases within the SRCC cohort (15/28, 54%) and in 26 cases within the control group (26/47, 55%). Regarding surgical margins in patients who underwent cystectomy (partial or radical), positive margins were reported in 5 of the 21 SRCC cases (5/21, 24%). In comparison, the control group showed positive surgical margins in 3 of the 24 cystectomy cases (3/24, 12.5%). Invasion of the abdominal wall, pelvic wall, or peritoneum was described in the clinical reports of 12 SRCC patients (12/28, 42.9%) and in 6 non-SRCC cases (6/47, 12.8%).

**Figure 2:**
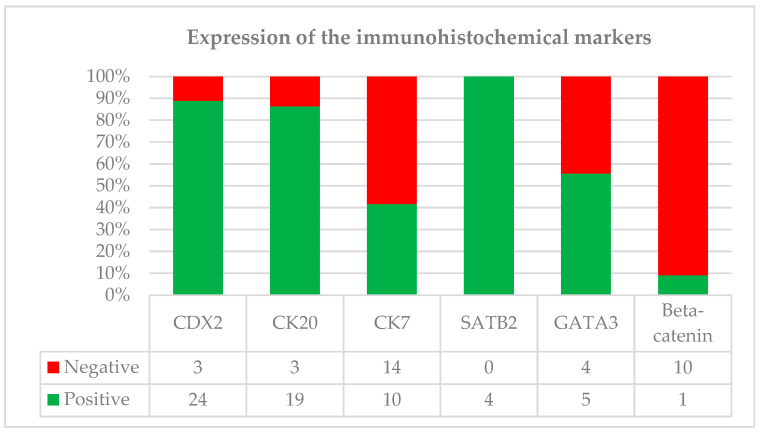
Immunohistochemical expression profile of urachal carcinoma, showing the number of positive (green) and negative (red) cases for key diagnostic markers (*p* > 0.05).

IHC analysis was performed on a subset of cases, revealing that the most consistently expressed markers were CK20 and CDX2 (Figure 2). Specifically, CK20 positivity was observed in 19 of 22 evaluated cases (19/22, 86.4%), while CDX2 expression was identified in 24 of 27 cases (24/27, 89%). CK7 expression was more variable, in 10 of 24 cases (10/24, 41.7%). β-Catenin staining was performed in 11 cases, 10 of which (10/11, 91%) demonstrated a membranous or cytoplasmic staining pattern. SATB2 was positive in all evaluated cases (4/4, 100%). Notably, GATA3, a marker typically associated with urothelial origin, showed focal or rare positivity in 5 of 9 evaluated cases (5/9, 55.6%). Other markers, such as p63, were consistently negative when assessed. Documented MMR evaluation was available for only 3 (3/75, 4%) patients in the database (two SRCC and one non-SRCC); in each instance, protein expression was intact.

### 3.3. Treatment Management and Survival Analysis

Surgical resections, including partial or radical cystectomies, were performed in 61 patients (61/75, 81.3%). Systemic chemotherapy was administered to 43 patients (43/75, 57.3%). Of these, 3 patients (3/43, 7%) received neoadjuvant therapy, 20 (20/43, 46.5%) received adjuvant treatment, and the remainder (20/43, 46.5%) were treated in a palliative setting. Radiotherapy was opted for in 12 cases (12/75,16%), often concurrently with chemotherapy. One patient (1/75, 1.3%) received immunotherapy.

Throughout the follow-up period, local recurrence in the bladder was noted in 3 cases, of which there were 2 SRCC patients (2/28, 7.1%) and 1 non-SRCC (1/47, 2.1%). Distant metastasis was documented in 46 cases overall (20/28, 71.4% in the SRCC cohort and 26/47, 55.3% in the control group), with the most common sites of metastasis being the lungs (n = 14), peritoneum (n = 14), bone (n = 13), liver (n = 9), distant lymph nodes (n = 8) and brain (n = 4).

**Figure 3:**
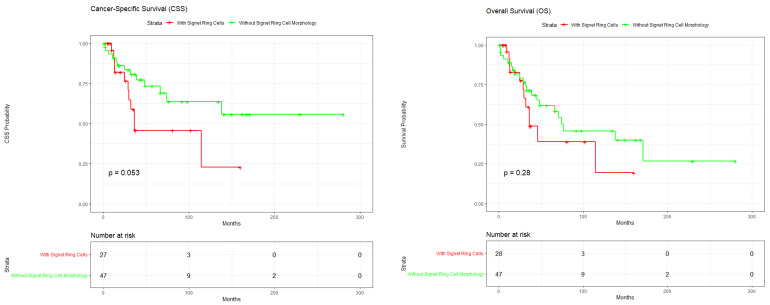
**Survival Analysis of the Urachal Carcinomas of the Bladder**. Multivariate Cox analysis for signet ring cell morphology showing OS (HR = 3.38; 95% CI: 1.22–9.33; *p* = 0.019) and CSS (HR = 3.15; 95% CI: 1.15–8.68; *p* = 0.026).

The median follow-up time for the entire cohort was 52 months (range: 1–280 months). The mean follow-up was 35.8 months for the SRCC group and 62.1 months for the control group. Regarding CSS, the 5-year survival rate was 39% for the SRCC cohort compared to 64% for the control group (*p* = 0.053). Overall Survival (OS) analysis showed no statistically significant difference between the two cohorts (*p* = 0.28) (Figure 3). In the multivariate Cox proportional hazards model, signet ring cell morphology was identified as an independent predictor of OS (HR = 3.38; 95% CI: 1.22–9.33; *p* = 0.019) and CSS (HR = 3.15; 95% CI: 1.15–8.68; *p* = 0.026).

## 4. Discussion

Urachal carcinoma is a rare malignancy with the signet-ring cell subtype showing a particularly aggressive course and poor prognosis. Here, we present a brief report on 28 cases of urachal SRCC we gathered from a combined medical database (BC Pathology and MD Anderson) as we compare clinicopathological features and survival patterns to those of urachal carcinoma cases without signet ring cells.

Most urachal neoplasms are invasive lesions, and often present similar symptoms to other urinary bladder lesions [28]. Hematuria was the predominant symptom identified in both the SRCC (n = 12) and non-SRCC (n = 19) patients, with no significant differences in the frequency reported for the two cohorts. Hematuria is the most common symptom of urachal carcinomas, represented in 58–82% of cases [6,8,28]. Less commonly, patients with urachal carcinomas can present dysuria, abdominal pain (12–14%), and mucinuria (10%), symptoms consistent with our findings [8,10,28]. Non-specific urinary symptoms like chronic urinary tract infections, pollakiuria, and pyuria can also occur, with approximately 8% of patients being asymptomatic [8]. As noted by Loizzo et al. [28], urachal carcinomas often remain asymptomatic until advanced stages. Sahu et al. [29] describe urachal SRCC as a clinically silent entity until advanced stages, presenting with atypical metastases.

Compared to other conventional urothelial carcinomas of the bladder, where the average age at diagnosis is 65 years, urachal SRCC affects slightly younger patients [30,31].

Patients from this study had a mean age at diagnosis 53 years, comparable to the control group (54 years) and consistent with established demographic trends in the literature [3,20,32,33]. While previous reports describe an age range for this rare subtype between 17 and 73 years [14,16], our cohort demonstrated a slightly broader distribution, with cases ranging from 28 to 82 years. The SRCC subtype shows a marked male predominance, with literature reporting up to 80% of cases occurring in men [20], confirmed also by the 75% male incidence in this SRCC cohort.

The staging of urachal carcinoma remains a subject of ongoing debate. The recent Dublin ISUP Consensus Conference [34] highlighted the limitations of the existing systems used to date: Sheldon [35], the Mayo system [36], the Ontario system [37], and the TNM/AJCC system [25]. Participants at the ISUP Consensus Conference supported moving away from the traditional systems and agreed in a majority (77%) that a new, modified TNM/AJCC system, specifically designed for urachal malignancies, would be the best approach [34]. To avoid any differences in reporting, we used the TNM/AJCC system, and observed a difference in the pT3 disease (perivesical fat invasion), with 61% of the SRCC group compared to 43% of controls. These findings mirror the pathological profile previously reported in the literature [12,13,14,16,18,19,21,38]. Applying the staging criteria proposed by Limonnik et al. (using the Surveillance, Epidemiology, and End Results—SEER database criteria) [39], we observed a 75% stage increase in the SRCC cohort. This high frequency of advanced-stage presentation was also reported in their findings, underlining that signet-ring cell morphology is an independent predictor of higher tumor stage [39].

The current diagnostic criteria for urachal carcinoma are still largely based on the clinical presentation and the location of the tumor: (1) location of the tumor in the dome/anterior wall; (2) epicenter of carcinoma in the bladder wall; (3) absence of widespread cystitis cystica/glandularis beyond the dome/anterior wall; and (4) absence of a known primary elsewhere [40]. However, morphological diagnosis of urachal adenocarcinoma represents a significant diagnostic challenge due to its histological similarities to pure bladder adenocarcinoma and metastatic colorectal carcinomas [2,10]. In this case, the usual IHC studies are of limited value.

The overall IHC analysis revealed 89% of cases expressed CDX2, and 86% were CK20-positive. About half of the cases expressed CK7 (42%) and GATA3 (56%). While CDX2, CK20, CK7, and GATA3 are supportive for the diagnosis of urachal carcinoma, their specificity is limited [2,28]. According to the literature, diagnosis of urachal bladder cancer is characterized by robust CK20 (97%) and CDX2 (90%) expression, while the lack of GATA3 expression and nuclear β-catenin (reported to be nuclear in 14% of cases) argues against a primary urothelial carcinoma with glandular differentiation or a secondary involvement of a colorectal carcinoma, respectively [2]. In urachal carcinomas, the expression of nuclear β-Catenin is rare and is also not often seen in pure bladder adenocarcinomas, even though it is identified in colorectal cancers [2,41]. The expression of nuclear β-Catenin can help in identifying urachal adenocarcinomas from bladder metastasis of colorectal adenocarcinomas, but it is not beneficial in differentiating urachal carcinomas from primary bladder adenocarcinomas [8,24]. When it comes to SRCC, markers such as PAS, CEA, and EMA show high sensitivity but remain non-specific due to expression overlapping with other signet-ring malignancies [42]. In addition to tissue markers, elevated serum levels of CEA, CA19-9, and CA125 are frequently observed and may assist in monitoring the disease [2,28]

Treatment options reported for the 75 patients included partial cystectomy as the dominant surgical approach (68%), and radical cystectomy in 15% of cases. This distribution aligns with the management trends described by Loizzo et al. [28] and Suartz et al. [32], who encourage bladder-sparing surgeries (partial cystectomy with en bloc resection of the umbilicus) as the standard of care for non-metastatic urachal carcinomas [28,43,44]. This surgical preference is further supported by a recent publication, which reported a 55% partial cystectomy rate and found no significant survival advantage for radical resections in urachal carcinoma cases [45]. While radical cystectomy is reserved for extensive tumors, various articles indicate no survival advantage over partial cystectomy if the specimen has negative margins [3,22]. Although lymph node dissection is required for accurate staging, its therapeutic benefit is still disputed [31,33].

For advanced or metastatic disease, regimens follow gastrointestinal protocols due to the biological resemblance to colorectal cancer [6,8,32]. 5-Fluorouracil (5-FU)-based combinations, such as FOLFOX (5-FU, leucovorin, oxaliplatin) or GemFLP, have demonstrated efficacy in first-line settings [29,46]. Emerging evidence supports the use of targeted therapies (e.g., EGFR inhibitors) and immunotherapy (e.g., atezolizumab) for patients with specific molecular profiles like KRAS mutations or MSI-high status [6,8]. Additionally, cytoreductive surgery combined with hyperthermic intraperitoneal chemotherapy (HIPEC) has granted long-term recurrence-free survival in selected cases with peritoneal metastases [8,28]. Across the entire study, adjuvant management was represented by chemotherapy (61%) and radiation (21%), whereas immunotherapy was reported in one case (1.3%).

Despite receiving standard-of-care resection options, the SRCC group experienced significantly higher recurrence rates (82%) compared to the non-SRCC group (53%) (*p* = 0.011). Moreover, 86% of SRCC patients received chemotherapy, yet the recurrence rate remained above 80%. This could mean that the SRCC subtype may be characterized by early micrometastatic spread and possible chemoresistance, as previously proposed by Chew et al. [42]. Chemoresistance and the aggressive nature of the disease are also corroborated by the findings of Sakthivel et al. [47], who reported that current systemic therapies do not confer a survival benefit for the SRCC variant. Their study supports our observations that the histologic subtype is a far more potent predictor of survival than the extent of surgical resection, as SRCC patients have significantly worse outcomes even after radical cystectomy. In this scenario, conventional therapeutic strategies may be insufficient for the management of urachal SRCC patients.

In a recent comparative analysis utilizing the National Cancer Database (NCDB), the SRCC variant proved to be significantly more aggressive, with patients showing a much shorter median OS compared to those with non-SRCC tumors (29.6 months vs. 79.0 months) [47]. Our own observed median survival times are consistent with these nationwide findings. Survival analysis revealed no statistically significant difference in OS between the two cohorts (*p* = 0.28). Published literature indicates that conventional urachal adenocarcinomas are associated with OS rates of approximately 43% to 61%, in line with the findings in our control group. On the other hand, the SRCC subtype is historically associated with poorer outcomes, with reported OS rates of approximately 22%, though our SRCC results proved a higher rate of 39% [8,20]. Published CSS estimates for urachal carcinoma range from 35% to 67%, which is consistent with the 64% rate observed in our non-SRCC group [28,31,48]. The multivariate Cox proportional hazards model analysis confirmed that signet ring cell morphology is a strong, independent predictor of worse outcomes, significantly increasing the risk for both OS (HR = 3.38; *p* = 0.019) and cancer-specific mortality (HR = 3.15; *p* = 0.026). These findings confirm the results from NCDB, which similarly found the SRCC subtype to be associated with a significantly increased risk of all-cause mortality (HR = 6.02) when compared to non-mucinous adenocarcinomas [3]. Despite its aggressive nature, urachal SRCC appears to have better survival outcomes than urothelial carcinoma of the bladder with the signet ring cell subtype, with one study mentioning a five-year survival of 44% for urachal SRCC versus only 15% for the bladder non-urachal primary with signet ring cell morphology [10].

Given the aggressive nature of urachal carcinoma, timely diagnosis and targeted treatment are critical for maximizing patient survival, as shown by the significant impact on survival rates. The limitation of this study is the fact that the collected patient database spans an extended time period, which introduces variability in surgical and systemic treatment protocols over time.

## 5. Conclusions

Due to the low incidence of signet ring cell adenocarcinoma of the urachus, the treatment and staging of these cancers is only just being discussed. Early diagnosis and staging, and an appropriate management plan, are essential to treating this aggressive variant of urinary malignancy. Radical cystectomy and partial cystectomy have shown success specifically in earlier stages, however in later stages, prognosis is poor.

## Figures and Tables

**Table 1:** Overview of the general demographic, clinical, and pathological features of the cohorts.

	Signet-Ring Cells (N = 28)	Non-SRCC Morphology (N = 47)	*p*-Value
**Age**	53 (14)	54 (14)	0.6
**Sex**			0.088
F	7 (25%)	21 (45%)	
M	21 (75%)	26 (55%)	
**Tumor Size (cm)**	3.48 (1.74)	3.23 (2.13)	0.5
**Procedure Type**			**0.037**
Biopsy	0 (0%)	7 (15%)	
Cystectomy	21 (75%)	24 (51%)	
TUR	7 (25%)	16 (34%)	
**Pathological Stage (pT)**			0.16
pT1	0 (0%)	4 (8.5%)	
pT2	3 (11%)	13 (27%)	
pT3	17 (61%)	20 (43%)	
pT4	4 (14%)	6 (13%)	
pTx	4 (14%)	4 (8.5%)	
**Metastasis at time of diagnosis**			1.0
Present	1	3	
Absent	27	44	

AJCC staging system 8th ed. [25].

## Data Availability

The data presented in this study are available on request from the corresponding author.
